# A Room Temperature ZnO-NPs/MEMS Ammonia Gas Sensor

**DOI:** 10.3390/nano12193287

**Published:** 2022-09-21

**Authors:** Ting-Jen Hsueh, Ruei-Yan Ding

**Affiliations:** Department of Electronic Engineering, National Kaohsiung University of Science and Technology, Kaohsiung 807, Taiwan

**Keywords:** nanoparticles, gas sensor, ZnO

## Abstract

This study uses ultrasonic grinding to grind ZnO powder to 10–20-nanometer nanoparticles (NPs), and these are integrated with a MEMS structure to form a ZnO-NPs/MEMS gas sensor. Measuring 1 ppm NH_3_ gas and operating at room temperature, the sensor response for the ZnO-NPs/MEMS gas sensor is around 39.7%, but the origin-ZnO powder/MEMS gas sensor is fairly unresponsive. For seven consecutive cycles, the ZnO-NPs/MEMS gas sensor has an average sensor response of about 40% and an inaccuracy of <±2%. In the selectivity of the gas, the ZnO-NPs/MEMS gas sensor has a higher response to NH_3_ than to CO, CO_2_, H_2_, or SO_2_ gases because ZnO nanoparticles have a greater surface area and more surface defects, so they adsorb more oxygen molecules and water molecules. These react with NH_3_ gas to increase the sensor response.

## 1. Introduction

Gas sensors are used in the petrochemical industry and manufacturing and to monitor environmental pollution as an early warning device to protect health. These toxic gases contain NO_x_, SO_x_, ammonia (NH_3_) and H_2_S. NH_3_ is one of the most widely produced chemicals, with annual production exceeding 100 million tons [1]. In addition to the semiconductor industry, NH_3_ is also widely used in the food industry, the refrigeration industry and clinical medicine [2]. NH_3_ is a colorless gas with a pungent odor that irritates the eyes and upper respiratory tract. In small concentrations, it affects human health. Therefore, it is very important to detect NH_3_.

In the last decade, standard air quality inspection stations and small gas sensors, such as infrared dots, catalytic beads, and photoionized and metal oxide semiconducting (MOS) sensors, have been the subject of many studies [3]. Recently, a polarization-insensitive design of a hybrid plasmonic waveguide to detect methane gas [4], using SmFeO_3_ material to detect acetylene, has also been reported [5]. However, for applications to protect human health on the go, gas sensors must be miniaturized, sensitivity must be increased, and power consumption and cost must be reduced. MOS sensors have been widely studied because of their small size, low cost and compatibility with silicon-based microelectronic devices. Semiconductor sensors with microelectromechanical systems (MEMS) are easily embedded in consumer devices and are suitable for wearable applications. These MOS materials include SnO_2_ [6], ZnO [7], CuO [8], La_2_O_3_ [9], CeO [10], and TiO_2_ [11].

ZnO is a chemically and thermally stable n-type MOS that features a 60-electronvolt exciton binding energy and a 3.2-electronvolt bandgap at room temperature [12], hence it is widely used in photodetectors, pH sensors and to detect toxic and combustible gases [13]. In recent years, MOS gas sensors have become more sensitive and power consumption has decreased. Nanostructures such as nanowires, nanoparticles and nanotubes are used to increase the sensitivity of sensors because, unlike body membrane and thin film sensors, nanostructured sensors have a greater response due to their large aspect ratio and high surface area to volume ratio [7].

To decrease power consumption, gas sensors must detect toxic gases at ambient temperature (~25 °C). Wang et al. reported a room-temperature gas sensor that has a ZnO nanorod/Au structure, and the sensitivity was 3.59 at 1 ppm NH_3_ [14]. Irajizad et al. reported a H_2_S gas sensor based on aligned ZnO nanorods with flower-like structures that operates at room temperature; and the sensor response was 581 at 5 ppm H_2_S [15]. Dong et al. reported a room-temperature ZnO nanorods NH_3_ gas sensor; the results indicated that the sensitivity is about 8 at 500 ppm NH_3_ [16].

This study produces ZnO nanoparticles (ZnO-NPs) using ultrasonic wave grinding technology and these are combined with a MEMS structure to form a ZnO-NPs/MEMS NH_3_ room temperature gas sensor. The fabrication of the sensor and the characteristics of the ZnO-NPs are discussed. The sensing properties of the ZnO NPs at room temperature for NH_3_ gas are also determined.

## 2. Materials and Methods

To fabricate ZnO nanoparticles, 0.5 g of zinc oxide powder, 4.1 g of alcohol and 1 mm 30 g of yttria-stabilized zirconia (YSZ) were mixed in a glass bottle. The zinc oxide powder was purchased from the Echo chemical Co., Ltd. (Tainan, Taiwan), and the purity is 99.7%. The glass bottle was then placed in a DELTA DC200 ultrasonic oscillator and a ZnO nanoparticle solution was produced after 14 days of grinding.

**Figure 1:**
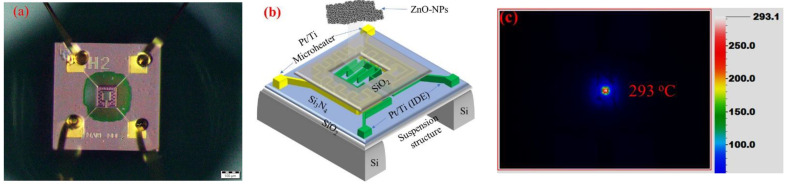
(**a**) The optical microscope, (**b**) the structure and (**c**) infrared thermal images of the ZnO-NPs/MEMS gas sensor.

The structure of the ZnO-NPs/MEMS gas sensor includes a MEMS structure and a sensing layer. The MEMS comprises a microheater, a sensing electrode, a dielectric layer of silicon nitride (Si_3_N_4_) and an isolation layer of silicon dioxide (SiO_2_) on a silicon (Si) suspension structure. Detailed fabrication methods have been reported [17], but the sensing layer is different to that of previous studies. Figure 1a shows the optical microscope image of the MEMS structure, and Figure 1b shows the schematic image of the ZnO-NPs/MEMS structure.

For this study, the ZnO nanoparticles solution was dropped directly onto the sensing electrode A snake-shaped micro heater surrounds the finger-shaped electrode. The ZnO-NPs/MEMS gas sensor was aged at about 293 °C for five days, using a microheater. Figure 1c shows an infrared (IR) thermal image of the ZnO-NPs/MEMS gas sensor. The sensing area is maintained at 293 °C, but the other area is close to room temperature because the suspension structure prevents thermal diffusion.

Scanning electron microscopy (SEM, STD PC, Jeol india pvt. Ltd., Tokyo, Japan) is used to observe the surface morphology of the ZnO nanoparticles. The crystallization of the ZnO nanoparticles was analyzed by X-ray diffraction (XRD, D8D Plus-TXS with CuKα radiation, Bruker, Billerica, MA, USA) at room temperature. The morphology and the structure of the ZnO-NPs were also measured using a transmission electron microscope (TEM, JEM-2100F, Jeol india pvt. Ltd., Tokyo, Japan). The gas sensor was placed into an airtight chamber. The volume of the chamber is about 8.8 L. The sensing properties of the sensor were measured using a source meter (Keithley 2400, Tektronix, Cleveland, Ohio, USA) that is connected to the sensing electrode and the results were recorded using a computer with LabVIEW software. During all measurements, the micro heater has no bias, and the voltage of the interdigitated electrodes (IDEs) is 5V. ZnO-NPs/MEMS gas sensor is operated at test chamber temperature (Note: the test chamber temperature is the same as the ambient room temperature). The ambient room temperature and relative humidity (RH) were about 23 °Cand 60%. In addition, ammonia gas is a mixture of 3% ammonia and 97% nitrogen (Yun Shan Gas Co., Tainan, Taiwan). The photoluminescence (PL) of the ZnO powder and ZnO-NPs was measured at room temperature using a 325 nm laser (HORIBA HR800, ISN, Cambridge, MA, USA).

## 3. Results and Discussion

**Figure 2:**
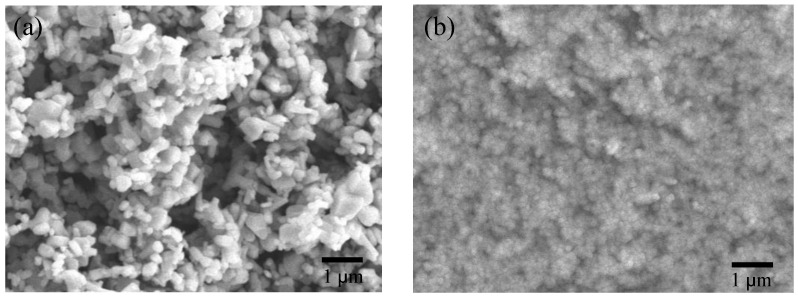
Top view SEM images of (**a**) origin-ZnO powder and (**b**) ZnO powder after ultrasonic grinding.

**Figure 3:**
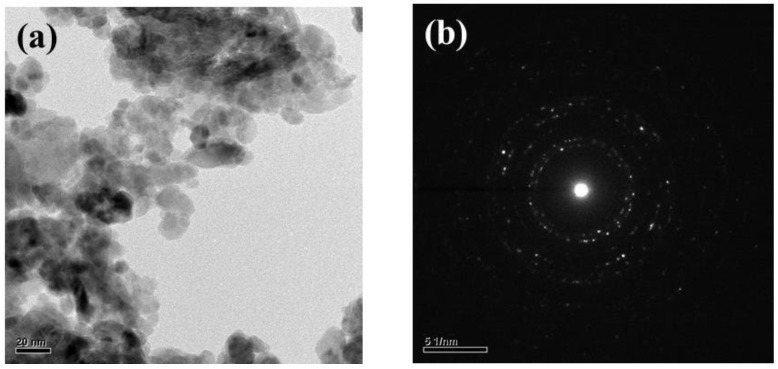
(**a**) The TEM image of the ZnO powder after ultrasonic grinding and (**b**) the selected area electron diffraction (SAED) image of the ZnO-NPs.

**Figure 4:**
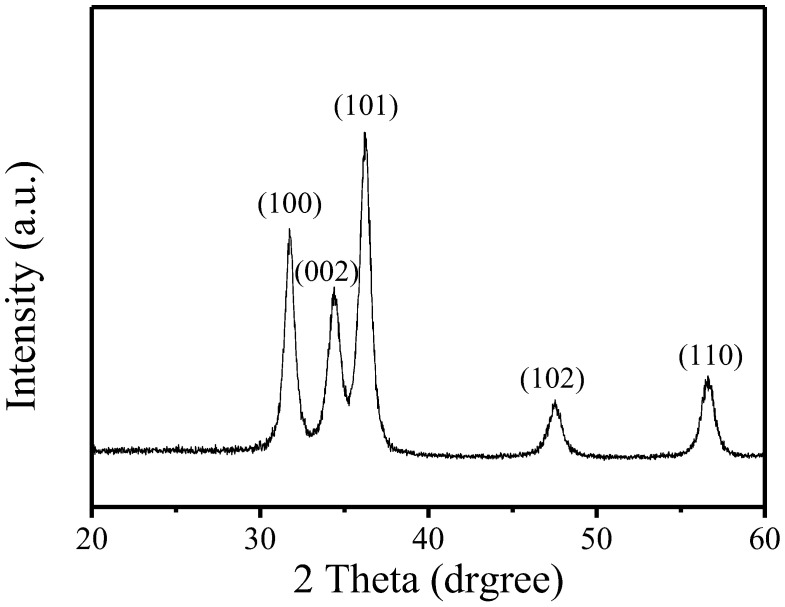
The XRD pattern for ZnO nanoparticles that are deposited on glass.

Figure 2a,b respectively show the top view SEM images of pure ZnO powder and ZnO powder after ultrasonic grinding. The size is not uniform and the pure ZnO powders are about 200 nm~1 µm, as shown in Figure 2a. ZnO powder is uniform after ultrasonic grinding and the particles are about 10–20 nm, as shown in the TEM image in Figure 3a. These results also show that ultrasonic grinding gives ZnO powder that is on the nanometer scale, so it is at least 20 times smaller. Figure 3b shows the selected area electron diffraction (SAED) image of the ZnO-NPs. The light spots on the map show multiple circular shapes so ZnO-NPs have a polycrystalline structure. Figure 4 shows the XRD pattern for ZnO nanoparticles that are deposited on glass. The value for 2θ is from 20° to 60°. The diffraction peaks are at 2θ = 31.75°, 34.48°, 36.39°, 47.68° and 56.65°. These peaks are typical of the planes of Wurtzite hexagonal ZnO and, respectively, correspond to rthe eflection of the (100), (002), (101), (102) and (110) planes (P63mc space group, a = 3.250 Å, c = 5.207 Å; JCPDS card no. 79-2205) [3]. In addition, using the Pseudo–Voigt function, it can be found that the full width at half maximum (FWHM) of the (100), (002), (101), (102), and (110) planes were about 0.73, 0.95, 0.82, 1.02, and 0.96. Therefore, using the Scherrer equation can be calculated the grain size of the (100), (002), (101), (102), and (110) planes were about 20.57 nm, 15.9 nm, 18.6 nm, 15.49 nm, and 17.2 nm.

**Figure 5:**
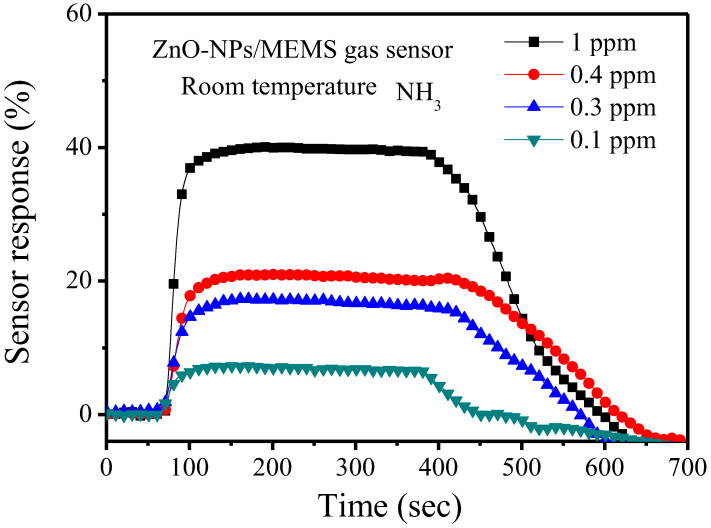
Sensor response for the ZnO-NPs/MEMS NH3 sensor at room temperature at various concentrations.

To measure NH_3_ gas, the sensor response is calculated as [(R_air_ − R_gas_)/R_air_ × 100%], where R_air_ is the resistance in air and R_gas_ is the resistance in the target gas: NH_3_. Figure 5 shows the sensor response for the ZnO-NPs/MEMS gas sensor. During the measurements, 1 ppm (parts per million, ppm), 0.4 ppm, 0.3 ppm, and 0.1 ppm NH_3_ were introduced into the airtight chamber at room temperature. Using this definition, the respective sensor response is around 39.7%, 21%, 17% and 6.9% for a concentration of NH_3_ gas of 1 ppm, 0.4 ppm, 0.3 ppm, and 0.1 ppm. The sensor response increases as the NH_3_ gas concentration increases. The response time and recovery time are not decreased at room temperature.

**Figure 6:**
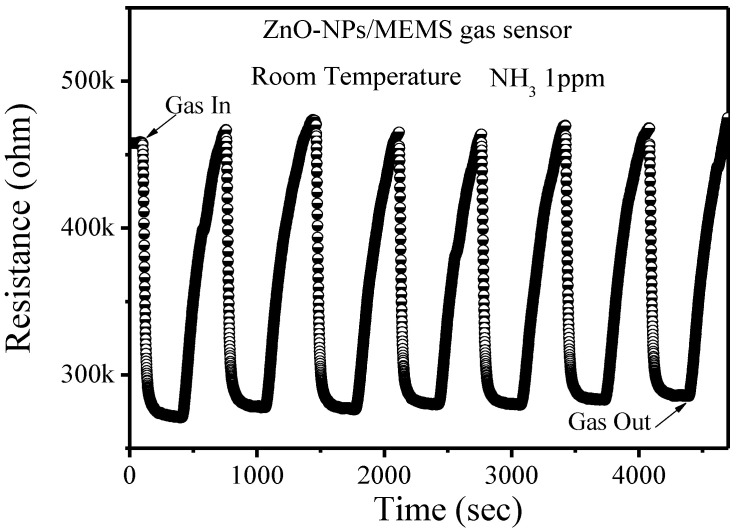
Response for a ZnO-NPs/MEMS NH_3_ sensor that is exposed to 1 ppm NH_3_ at room temperature.

**Figure 7:**
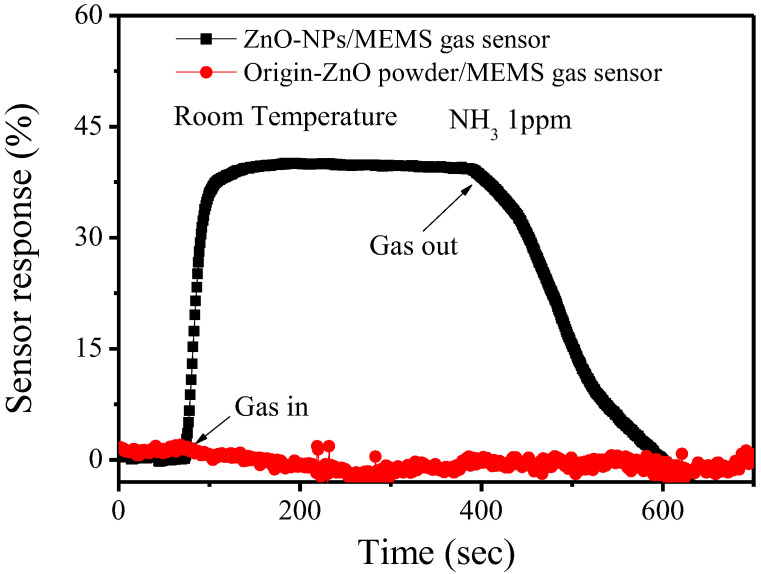
The sensor response for the origin-ZnO powder/MEMS gas sensor and the ZnO-NPs/MEMS gas sensor.

Figure 6 shows the stability and the reproducibility of the ZnO-NPs/MEMS gas sensor. This experiment used seven cycles for the ZnO-NPs/MEMS gas sensor. Each cycle involved injecting NH_3_ gas for 5 min and then exhausting the gas for 5 min. Figure 6 shows the switching sensor response for a ZnO-NPs/MEMS gas sensor that is exposed to 1 ppm NH_3_ gas at room temperature. When the NH_3_ gas is injected and exhausted, the resistance in the ZnO-NPs decreases and increases. Using the same definition, the average sensor response is about 40%, with an inaccuracy of <±2%. These results also show that the response of the fabricated sensor is stable and reversible. Figure 7 shows the sensor response for the origin-ZnO powder/MEMS gas sensor and the ZnO-NPs/MEMS gas sensor. The origin-ZnO powder/MEMS gas sensor is almost unresponsive. This result confirms that the ZnO-NPs/MEMS gas sensor senses low NH_3_ concentrations at room temperature.

**Figure 8:**
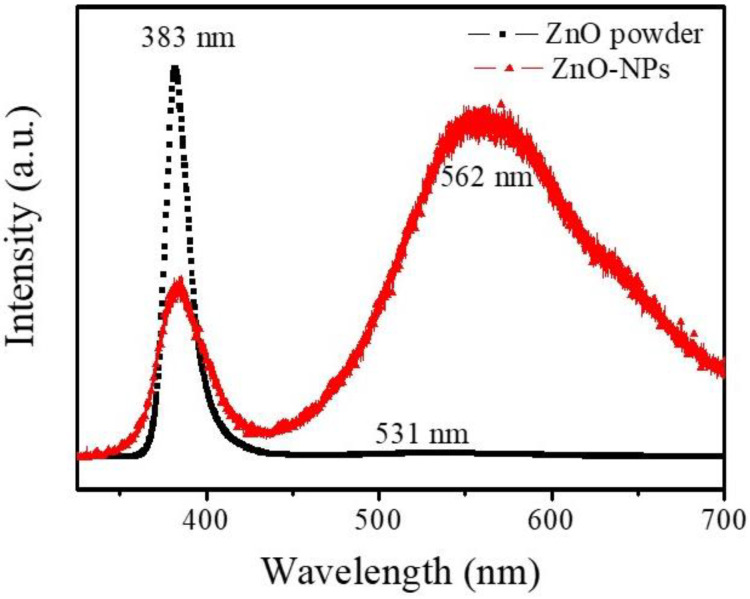
The room temperature photoluminescence spectrum for origin-ZnO powder and ZnO-NPs on glass.

In terms of the sensing mechanism for the room temperature ZnO-NPs/MEMS gas sensor, the material defects of the ZnO-NPs must be considered. Figure 8 shows the room temperature PL spectra for origin-ZnO powder and ZnO-NPs on glass. The PL spectra for the origin-ZnO powder clearly show that a sharp strong peak is located at around 383 nm. This UV emission peak is attributed to the near band-edge (NBE) emissions of ZnO (exciton transition) [18]. The PL spectra for ZnO-NPs are different to that for origin-ZnO powder. In addition to the NBE emissions, the ZnO-NPs also have a broad peak at around 562 nm. This is the deep level emission of ZnO (green–yellow band). This emission band is attributed to ionized oxygen vacancies (V_O_^+^), oxygen interstitials (O_i_) and neutral oxygen vacancies (V_O_) [18]. The source of these deep-level emissions is ZnO surface defects that are caused by the ultrasonic grinding process. Therefore, when a reducing gas (NH_3_) is injected, ZnO-NPs are more responsive than the origin-ZnO powder, as shown in Figure 7. In addition, these ZnO surface defects also provided lower NH_3_ concentration sensing, as shown in Figure 5.

**Figure 9:**
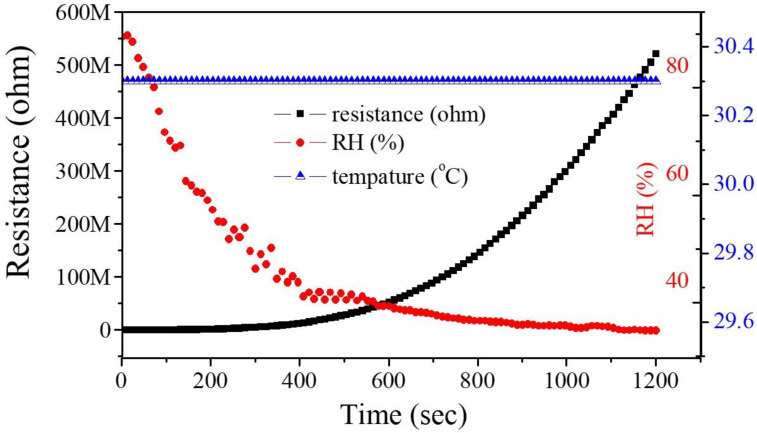
The effect of ambient temperature and humidity on the ZnO-NPs/MEMS gas sensor.

Oxygen molecules play an important role in the sensing mechanism of MOS gas sensors. When O_2_ is attached to the MOS surface, O_2_ captures electrons on the MOS surface to form oxygen ions (O_2_^−^, O^−^, or O^2−^) and the resistance of the n-type material (such as ZnO) increases and the resistance of p-type decreases. When the reducing gas (such as CO, NH_3_) is injected, it reacts with oxygen ions, and captured electrons return to the material so the resistance of the n-type material decreases, as shown in Figure 6. During gas sensing, the production of chemisorbed oxygen species is largely dependent on temperature. At lower temperatures (<150 °C) O_2_^−^ is chemisorbed and at higher temperatures, O^−^ is chemisorbed faster by O^2−^ [19,20,21]. The ZnO-NPs/MEMS gas sensor for this study operates at room temperature so the ZnO-NPs/MEMS gas sensor is affected by ambient temperature and humidity. Figure 9 shows the effect of ambient temperature and humidity on the ZnO-NPs/MEMS gas sensor. During the experiment, the RH decreases from ~90% to ~35% and the chamber temperature is 30.3 °C. The results show that the sensor’s resistance increases when humidity decreases. For a humidity of less than 40%, the sensor’s resistance increases significantly because water molecules are chemisorbed onto the surface of the ZnO-NPs when the sensor is in a low-humidity environment (<40%). The adsorption of the water molecules is not continuous, hence charges are hopped by protons (H^+^) [22,23]. In a medium-humidity environment (about 40%~75%), water molecules are chemisorbed and physisorbed onto the surface of the ZnO-NPs so physical adsorption of the water layer plays a leading role during the charge transfer [22,23]. In a high humidity environment (>75%), charge transport occurs in the physisorbed water layer. In this study, ammonia gas is measured in a medium-humidity environment, hence oxygen ions react with ammonia on the surface of the ZnO-NPs and water ions participate in the reaction. The reaction between oxygen ions and ammonia is written as [24]:

4NH_3_ + 3O_2_^−^ → 2N_2_ + 6H_2_O + 3e^−^ (1)

Physisorbed H_2_O also reacts with NH_3_ to produce NH_4_OH [25]. Therefore, if NH_4_OH is on the surface of the ZnO-NPs, it reacts with adsorbed O_2_ to generate nitrogen oxide gas [26], as:

4NH_4_OH + 7O_2_^−^ → 4NO_2_ + 10H_2_O + 15e^−^ (2)

These oxidation and reduction reactions enable the sensor to rapidly respond and recover.

**Figure 10:**
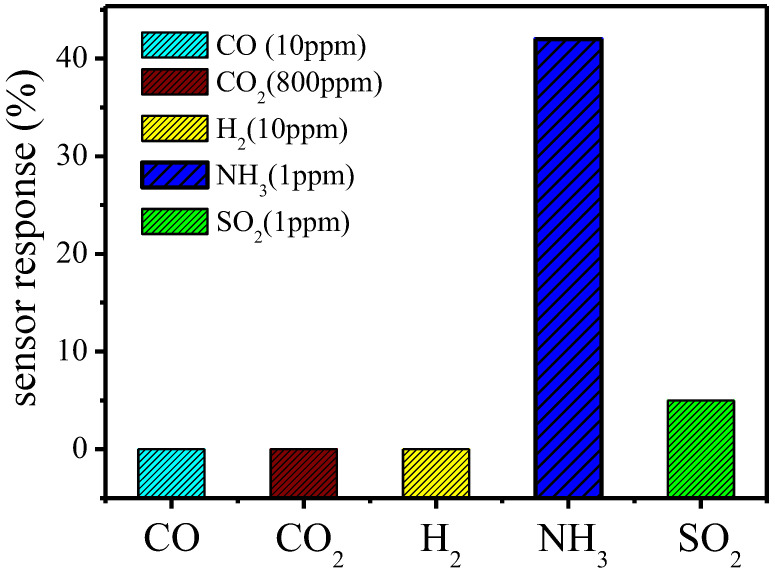
Response to CO, CO_2_, H_2_, NH_3_ and SO_2_ for a ZnO-NPs/MEMS gas sensor at room temperature.

Figure 10 shows the CO, CO_2_, H_2_, NH_3_, and SO_2_ responses for the ZnO-NPs/MEMS gas sensor at room temperature and ~60% RH. The sensor response for the ZnO-NPs/MEMS gas sensor is not significant when CO, CO_2_ and H_2_ are injected at approximately 10 ppm, 800 ppm and 10 ppm. There is a 5.3% sensor response if the sensor is in a 1 ppm SO_2_ atmosphere. These results show that the ZnO-NPs/MEMS gas sensor has a higher selectivity for NH_3_ gas.

## 4. Conclusions

Ultrasonic grinding is used to fabricate nanosized ZnO particles. The ZnO nanoparticles are combined with a MEMS structure to form a ZnO-NPs/MEMS gas sensor. TEM analysis shows that the ZnO nanoparticles are about 10 nm. To measure the gas sensor, the sensors were operated at room temperature. For a concentration of NH_3_ gas of 1 ppm, 400 ppb, 300 ppb and 100 ppb, the respective sensor response is around 39.7%, 21%, 17% and 6.9%. To measure the stability and reproducibility, the ZnO-NPs/MEMS gas sensor is subjected to seven consecutive cycles. The average sensor response is about 40% (1 ppm) with an inaccuracy of <±2%. The origin-ZnO powder/MEMS gas sensor is almost unresponsive. These results confirm that this study produces a room temperature ZnO-NPs/MEMS gas sensor that senses low NH_3_ concentrations.

## Data Availability

Not applicable.
